# Supplementary material for: Cell Sheets Restore Secretory Function in Wounded Mouse Submandibular Glands
Source: Cells. 2020 Dec 9;9(12):2645. doi: 10.3390/cells9122645 (PMC7763220; doi:10.3390/cells9122645)
Supplement: Supplementary file 1 [file cells-09-02645-s001.pdf]

cells

Supplementary Materials

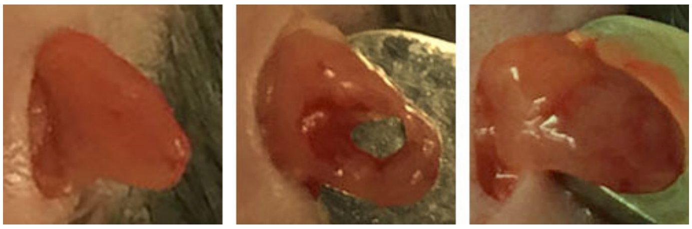

Figure 1. Cell sheets can be directly transplanted and attached to the wound site. Adapted with permission from [1] Copyright © 2019, npj Regenerative Medicine.

References

1. Nam, K.; Kim, K.; Dean, S.M.; Brown, C.T.; Davis, R.S.; Okano, T.; Baker, O.J. Using cell sheets to regenerate mouse submandibular glands. *NPJ Regen Med* **2019**, *4*, 16, doi:10.1038/s41536-019-0078-3.

Publisher's Note: MDPI stays neutral with regard to jurisdictional claims in published maps and institutional affiliations.

© 2020 by the authors. Licensee MDPI, Basel, Switzerland. This article is an open access article distributed under the terms and conditions of the Creative Commons Attribution (CC BY) license (http://creativecommons.org/licenses/by/4.0/).
